# Adjuvant Therapy in Pancreatic Ductal Adenocarcinoma—Past, Present, and Future

**DOI:** 10.3390/cancers18172754

**Published:** 2026-08-25

**Authors:** Andy Tran, Tejeshwar Jain, Naomi Fei, Vikas Dudeja

**Affiliations:** 1 Department of Surgery, University of Iowa, 1509 Colloton Pavilion (JCP), 200 Hawkins Drive, Iowa City, IA 52242, USA; 2 Holden Comprehensive Cancer Center, University of Iowa, Iowa City, IA 52242, USA; 3 Department of Surgery, University of Alabama at Birmingham, Birmingham, AL 35294, USA; 4 Division of Hematology, Oncology and Blood & Marrow Transplantation, University of Iowa, Iowa City, IA 52242, USA

**Keywords:** pancreatic cancer, pancreatic adenocarcinoma, adjuvant therapy, chemotherapy, radiation, immunotherapy

## Abstract

Pancreatic ductal adenocarcinoma (PDAC) remains one of the most lethal malignancies despite advances in surgical techniques and systemic therapy. Although surgical resection offers the only potential for cure, recurrence is common, underscoring the critical role of multimodal treatment. Over the past two decades, numerous clinical trials have cemented adjuvant chemotherapy as a cornerstone of multimodal management in resected PDAC, with progressive evolution from single-agent regimens to modern multiagent regimens. In contrast, the role of chemoradiation remains debated, with the body of evidence demonstrating conflicting results. Ongoing clinical trials are expected to elucidate optimal treatment modalities. Emerging targeted therapies, biomarker-directed treatment approaches, and immunotherapeutic strategies also hold promise for improving outcomes in select patient populations. This narrative review summarizes the landmark trials that have shaped adjuvant therapy, examines the evolution of adjuvant treatment over time, reviews current management guidelines, and highlights promising emerging therapeutic directions likely to influence the management of localized pancreatic cancer.

## Simple Summary

Pancreatic cancer is one of the deadliest cancers and carries a short life expectancy. Surgery is the only possible cure, but even when tumors are successfully removed, they often recur. To lower this risk, patients typically receive additional treatment, usually chemotherapy, after surgery. Over the past several decades, many studies have shown that giving chemotherapy after surgery helps patients live longer, and treatment regimens have steadily improved. However, there are still unanswered questions like whether adding radiation after chemotherapy or giving chemotherapy before surgery will help patients live longer. On top of answering these questions, scientists are investigating new drugs and drug combinations to target genetic mutations, harness the body’s immune system, or break down the dense supportive tissue that helps protect the tumor. This review discusses the most important studies that have shaped cancer treatment, describes current treatment recommendations, and highlights promising new directions in managing this disease.

## 1. Introduction

Pancreatic ductal adenocarcinoma (PDAC) is a highly lethal malignancy, with a 5-year overall survival of only 13% across all stages, making it the 3rd leading cause of cancer-related deaths [1]. The poor prognosis of PDAC is due, in part, to its clinically occult nature, which often results in diagnosis at an advanced stage, with more than half of patients presenting with metastatic disease [2]. Though advances in systemic therapies have improved outcomes, surgery remains the only potentially curative treatment, and for patients with resectable disease, surgery combined with systemic chemotherapy offers the best chance for long-term survival. However, even among patients who undergo resection, 5-year survival remains poor at 17% [3].

Over the past two decades, a multitude of trials have evaluated the role of adjuvant therapy, ultimately elucidating the critical role of multimodal therapy in the treatment of resectable PDAC. In this review, we will discuss the current body of evidence regarding adjuvant therapy and highlight the landmark clinical trials that have shaped current treatment strategies.

## 2. Adjuvant Chemotherapy

The ESPAC-1 (European Study Group of Pancreatic Cancer) trial, initially published in 2001, was the first large-scale clinical trial to demonstrate a significant survival benefit of adjuvant chemotherapy in pancreatic cancer patients [4]. The study utilized a 2 × 2 factorial design to randomize patients who had undergone curative-intent resection into four treatment groups: observation alone; chemotherapy alone (5-FU and leucovorin); chemoradiation alone (5-FU bolus with split-course radiation); and chemotherapy plus chemoradiation. With respect to adjuvant chemotherapy, the study demonstrated that median overall survival (mOS) was prolonged in those who received adjuvant chemotherapy when compared to those randomized into the observation arm (20.1 vs. 15.5 months, *p* = 0.009) [4]. The 5-year survival rate amongst those in the chemotherapy arm was 21%, compared to 8% amongst those in the observation-only group. Intriguingly (as described below), this trial suggested that patients in the chemoradiation group (with or without addition of adjuvant 5-FU/Leucovorin) fared worse than those in groups without chemoradiation (refer to Table 1 for a complete summary of landmark adjuvant chemotherapy trials).

Building on the reports of favorable outcomes with gemcitabine when compared with 5-FU-based chemotherapy in advanced PDAC [15], CONKO-001 (Charite Onkologie 001) [16] explored the role of adjuvant gemcitabine in the treatment of PDAC. In this study, patients who had undergone curative resection of PDAC were randomized to either observation alone or to six months of gemcitabine. CONKO-001 demonstrated that adjuvant treatment with gemcitabine led to improvement in disease-free survival (DFS) (DFS 13.4 months in the treatment group vs. 6.9 months in the observation group, *p* < 0.001) [16]. On analysis of long-term data, significantly prolonged median overall survival was also observed in the gemcitabine group (22.8 vs. 20.2 months, *p* = 0.01) compared to observation alone [5].

Similar to CONKO-001, JSAP-02 (Japanese Study Group of Adjuvant Therapy for Pancreatic Cancer) explored the role of adjuvant gemcitabine in the treatment of PDAC. One key difference between these studies was that CONKO-001 treated patients for 6 cycles compared to 3 cycles in JSAP-02. JSAP-02 demonstrated improvements in disease-free survival (DFS) (11.4 vs. 5.0 months, *p* = 0.01) but was unable to demonstrate statistically significant improvements in median overall survival (22.3 vs. 18.4 months, *p* = 0.19) [6]. Key differences between the two studies that may have contributed to the lack of significant differences in overall survival in JSAP-02 may be the shorter duration of treatment, shorter follow-up period, and a smaller study size in JSAP-02. Nonetheless, these studies established adjuvant gemcitabine monotherapy as standard of care at that time.

Further reinforcing the role of adjuvant gemcitabine in resected PDAC, RTOG (Radiation Therapy Oncology Group) 9704 directly compared the addition of gemcitabine or fluorouracil to adjuvant chemoradiation with fluorouracil. The study randomized patients with PDAC who had undergone gross resection of their disease to receive either gemcitabine or fluorouracil for 3 weeks before and 12 weeks after fluorouracil-based chemoradiation. Initial analysis of those with pancreatic head tumors showed a trend towards improved mOS with gemcitabine (20.5 vs. 16.9 months, *p* = 0.09), which reached statistical significance on multivariable analysis (*p* = 0.05) [17]. The five-year analysis confirmed a trend toward improved outcomes in the gemcitabine group (mOS 20.5 vs. 17.1 months, *p* = 0.08) [7]. Though the study’s primary endpoint did not reach significance, the trend toward improved survival with gemcitabine compared to fluorouracil was consistent with contemporary data demonstrating improved survival following adjuvant gemcitabine compared to observation and solidified gemcitabine’s value in resected pancreatic cancer.

Akin to RTOG 9704, ESPAC-3 compared the efficacy of 5-FU and gemcitabine in the adjuvant setting [8]. Notably, RTOG 9704 included chemoradiation in both study arms. At this time, the role of adjuvant chemoradiation in PDAC had become a point of debate. With the results of ESPAC-1 not only suggesting no benefit but potentially harm with adjuvant chemoradiation, it was deliberately excluded in ESPAC-3. The results of this trial demonstrated that 5-FU plus leucovorin and gemcitabine were equivalent in terms of overall survival (mOS 23.0 months with 5-FU/Leucovorin vs. 23.6 months with gemcitabine, *p* = 0.39). However, patients in the gemcitabine group had significantly fewer adverse reactions [8]. About 14% of patients in the 5-FU group experienced treatment-related serious adverse effects compared to 7.5% of patients in the gemcitabine group (*p* < 0.001). This study established both regimens as acceptable adjuvant therapies, though gemcitabine appeared to offer a more tolerable toxicity profile.

With the solidification of gemcitabine as the preferred single-agent chemotherapy in resected PDAC, Japanese investigators conducted JASPAC-01 (Japan Adjuvant Study Group of Pancreatic Cancer), a trial comparing gemcitabine to S-1, an oral fluoropyrimidine, in the adjuvant setting [10]. Conducted in 33 hospitals across Japan, the study demonstrated clear superiority of S-1 over gemcitabine with significantly improved median overall survival (46.5 vs. 25.5 months, *p* < 0.0001) [10]. Based on these results, S-1 became the standard first-line agent in resected disease in Japan [18]. Despite the striking results, adjuvant S-1 did not become the standard of care globally due to metabolic variations between Asian and non-Asian populations. Due to polymorphic differences in CYP2A6 leading to higher exposure to toxic metabolites, non-Asian populations experience greater toxicity at equivalent doses [19,20,21]. Consequently, the results of JASPAC-01 were not validated in non-Asian populations. Rather, Western investigators went on to explore multiagent regimens.

With the results of ESPAC-3 demonstrating the equivalence of gemcitabine and fluorouracil as single-agent therapies, investigators began exploring the effectiveness of multiagent regimens. CONKO-005 explored the benefit of erlotinib, an oral quinazoline derivative that inhibits epidermal growth factor-related tyrosine kinase, in conjunction with gemcitabine. While gemcitabine plus erlotinib had previously been shown to confer modest improvement in overall survival compared to gemcitabine alone in unresectable PDAC [22], CONKO-005 failed to demonstrate a statistically significant benefit in disease-free or overall survival with the addition of erlotinib to gemcitabine in resected disease (mOS 24.5 months for combination vs. 26.5 months in gemcitabine alone, *p* = 0.61) [11].

Building on the success of gemcitabine plus nab-paclitaxel in metastatic PDAC [23], the APACT (Adjuvant Pancreatic Adenocarcinoma Clinical Trial) evaluated this combination in resected PDAC, comparing its efficacy to that of gemcitabine alone [12]. The study’s primary endpoint was independently assessed disease-free survival, which was not met (19.4 vs. 18.8 months for nab-paclitaxel plus gemcitabine vs. gemcitabine alone, *p* = 0.18). However, investigator assessment demonstrated significantly improved disease-free survival (16.6 vs. 13.7 months, *p* = 0.02) as well as improved median overall survival (41.8 vs. 37.7 months, *p* = 0.009) with the combination regimen. This discrepancy has been attributed to differences in imaging interpretation and timing of assessments. Despite not meeting its primary endpoint, the trial suggests a modest overall survival benefit with adjuvant nab-paclitaxel plus gemcitabine and reinforces the strategy of cytotoxic intensification. However, nab-paclitaxel plus gemcitabine is not listed as a preferred therapy for adjuvant therapy in NCCN guidelines [24].

ESPAC-4 was the first trial to definitively demonstrate superiority of a gemcitabine-based regimen over gemcitabine monotherapy in the adjuvant setting. This study compared gemcitabine alone to gemcitabine plus capecitabine, an oral fluoropyrimidine. The researchers found significantly improved median overall survival with the addition of capecitabine (28.0 vs. 25.5 months, *p* = 0.03) [9]. Importantly, the study did not show an increased risk of treatment-related serious adverse effects with the combination regimen. Long-term follow-up data continued to demonstrate improved median overall survival with the combination therapy (31.6 vs. 28.4 months, *p* = 0.03) [13]. Even more, subgroup analysis of patients with poor performance status, who would not have qualified for the PRODIGE 24 trial (see below), continued to demonstrate survival advantage with the combination regimen (mOS 25.9 vs. 20.7 months, *p* = 0.04). These results led to the incorporation of gemcitabine plus capecitabine as a preferred chemotherapy regimen for adjuvant treatment of resected pancreatic cancer [24].

Departing from contemporary trials which explored gemcitabine-based regimens, PRODIGE 24 (Partenariat de Recherche en Oncologie Digestive)/CCTG PA6 (Canadian Cancer Trials Group Pancreatic Adenocarcinoma) explored the role of modified FOLFIRINOX (mFOLFIRINOX) as adjuvant therapy for resected PDAC in fit patients (ECOG performance status 0–1) [25]. Compared to standard FOLFIRINOX, mFOLFIRINOX omits the 5-FU bolus at the start of each cycle and includes a dose reduction of irinotecan. This study found significantly improved median overall survival with mFOLFIRINOX compared to gemcitabine monotherapy (54.4 vs. 35.0 months, *p* = 0.003) [25], which persisted in long-term analysis (53.5 vs. 35.5 months, *p* = 0.001) [14]. However, patients in the mFOLFIRINOX group more frequently experienced significant treatment-related adverse events (75.9% vs. 52.9%, *p* < 0.001). Notably, subsequent re-analysis of this cohort demonstrated that R1 resection margin status was an independent predictor of poorer DFS in the gemcitabine arm (HR 1.95, *p* = 0.005) but not the mFOLFIRINOX group (HR 1.46, *p* = 0.11) [26], suggesting that mFOLFIRINOX may mitigate the adverse prognostic impact of positive resection margins. This study reported the longest overall survival outcomes of any comparable trial and led to a paradigm shift in the treatment of resected pancreatic cancer. mFOLFIRINOX is now recommended as a preferred regimen in adjuvant treatment of PDAC in patients who can tolerate the therapy [24].

## 3. Contemporary Adjuvant Chemotherapy for Pancreatic Cancer in 2026

Contemporary adjuvant chemotherapy for pancreatic cancer consists primarily of modified FOLFIRINOX (mFOLFIRINOX) or gemcitabine plus capecitabine, both of which are designated as preferred, category 1 regimens by the NCCN. Thus, treatment selection is largely guided by patient performance status, comorbidities, and anticipated treatment tolerance. Notably, enrollment in PRODIGE 24 was completed before the publication of ESPAC-4, precluding a direct comparison between mFOLFIRINOX and gemcitabine plus capecitabine. Secondary analyses of PRODIGE 24 provided important insights into the impact of pathologic risk factors on treatment benefit. R1 resection was associated with worse disease-free survival (DFS) among patients treated with gemcitabine but not among those receiving mFOLFIRINOX. Furthermore, the overall survival benefit of mFOLFIRINOX was maintained across adverse pathologic subgroups, suggesting that intensified multiagent therapy may mitigate the adverse prognostic impact of high-risk pathologic features [26]. In contrast, long-term follow-up of ESPAC-4 suggested that the benefit of gemcitabine plus capecitabine may be greater among patients with more favorable pathologic features. Although combination therapy improved median overall survival, the benefit was statistically significant among patients with node-negative disease (HR 0.63, *p* = 0.04) but not among those with node-positive disease (HR 0.90, *p* = 0.23) [13].

Although no head-to-head trial has directly compared the toxicity profiles of mFOLFIRINOX and gemcitabine plus capecitabine, grade 3–4 adverse events were more frequent among patients treated with mFOLFIRINOX in the PRODIGE 24 trial. In PRODIGE 24, grade 3–4 adverse events occurred in 75.9% of patients treated with mFOLFIRINOX versus 52.9% with gemcitabine, while in ESPAC-4, grade 3–4 events occurred in 63% of the gemcitabine plus capecitabine arm compared to 54% in the gemcitabine group. Although cross-trial comparisons have inherent limitations, the higher incidence of adverse events with mFOLFIRINOX is notable, particularly given that the PRODIGE 24 trial enrolled a substantially fitter patient population. Interestingly, a larger proportion of patients receiving mFOLFIRINOX in PRODIGE 24 completed all planned cycles (66.4% vs. 79.0% with gemcitabine monotherapy) than did patients receiving gemcitabine plus capecitabine in ESPAC-4 (54% vs. 65% with gemcitabine alone). Again, conclusions regarding comparative tolerance cannot be made given the cross-trial nature of this data; however, this discrepancy may partially reflect the highly selected PRODIGE 24 population, which was restricted to ECOG 0–1, age ≤ 79 years, and postoperative CA 19-9 < 180 U/mL. Real-world observational data demonstrate similar completion rates for gemcitabine plus capecitabine compared to gemcitabine alone (69.5% vs. 62.7%, *p* = 0.11), suggesting the addition of capecitabine does not compromise tolerability [27]. In contrast, observational data on mFOLFIRINOX tolerability are highly variable and heavily dependent on cohort selection, although the regimen is generally regarded as more toxic and associated with lower completion rates compared to gemcitabine-based regimens. Even when all planned cycles are delivered, mFOLFIRINOX frequently requires dose reductions or discontinuation of individual agents, commonly because of the cumulative and potentially permanent oxaliplatin-induced peripheral neuropathy. In PRODIGE-24, oxaliplatin more frequently required dose reduction than any other agent and was discontinued after a median of 9 cycles. Similarly, a real-world cohort reported an 84% completion rate for mFOLFIRINOX, yet 67% required early discontinuation of oxaliplatin, most frequently because of neuropathy, after a median of 10 doses [28]. The differences in adverse event rates and treatment completion between mFOLFIRINOX and gemcitabine plus capecitabine are particularly important, as evidence consistently demonstrates an association between completion of the planned course of chemotherapy and improved outcomes. Notably, in PRODIGE 24, completion of adjuvant therapy was independently associated with improved overall survival [14,29].

Beyond selection of the appropriate chemotherapy regimen, the timely initiation of adjuvant therapy is also an important consideration. The NCCN recommends initiating adjuvant chemotherapy once the patient has adequately recovered from surgery, ideally within 12 weeks of resection. The emphasis, however, should be placed on adequate recovery. Both early and late initiation of adjuvant chemotherapy may be disadvantageous [30]. In an ancillary analysis of JASPAC 01, initiation both later than 8 weeks and earlier than 6 weeks following surgery was associated with shorter survival [31]. The reduced survival observed among patients who initiated chemotherapy early may reflect differences in treatment tolerance rather than reduced treatment efficacy. Indeed, earlier initiation of adjuvant therapy has been associated with a lower likelihood of completing the planned course of chemotherapy [32]. Delayed initiation, on the other hand, may reflect postoperative complications and potentially allow micrometastatic disease to proliferate unabated [33]. Given the observational nature of these studies and varied definitions of timely chemotherapy initiation, it is difficult to derive definitive conclusions. What is clear, however, is that completion of chemotherapy greatly impacts survival, more so than precise initiation timing [29].

Taken together, these data support a pragmatic approach to adjuvant therapy selection, balancing treatment efficacy with patient tolerance, pathologic risk, and comorbidities. mFOLFIRINOX should be considered for patients with good performance status (ECOG 0–1) who are able to tolerate an intensive multiagent regimen, particularly those with high-risk pathologic features. Conversely, gemcitabine plus capecitabine may represent a more appropriate option for patients in whom treatment tolerance is of greater concern. Emerging genomic analysis from PRODIGE 24 suggests that KRAS-wild-type tumors may be associated with poor response to mFOLFIRINOX, raising the prospect of biomarker-aided regimen selection in the future, although the investigators emphasize that this finding remains hypothesis-generating and should not yet alter clinical practice [34].

## 4. Adjuvant Chemoradiation

While the role of adjuvant chemotherapy is well established and has become standard of care, the role of radiation and chemoradiation remains uncertain. Given that locoregional recurrence accounts for 40–60% of recurrent disease [35], and that early recurrence [36] as well as positive resection margins [37] correlate with decreased survival, the addition of radiation therapy (RT) to adjuvant treatment regimens aims to improve outcomes by augmenting locoregional control. The role of adjuvant therapy of any kind was first investigated in a small randomized clinical trial conducted by the Gastrointestinal Tumor Study Group (GITSG 9173) in 1985 [38]. In this study, 43 patients who had undergone curative-intent resection for PDAC were randomized to observation versus adjuvant chemoradiation (CRT) followed by weekly maintenance 5-FU infusions for two years. Those who received adjuvant therapy had significantly longer overall survival compared to those who did not (mOS 21 vs. 11 months, *p* = 0.03). Based on this study, adjuvant chemoradiation became the standard of care over the subsequent years. However, the study had several shortcomings, including a small sample size, use of split-course radiation, a lower overall radiation dose, and a lack of quality control for radiation dosing. Additionally, the intervention arm included both chemotherapy and radiation treatments; therefore, it was unclear which modality contributed to the improved outcomes (refer to Table 2 for a complete summary of landmark adjuvant chemoradiation trials) [39].

The European Organization for Research and Treatment of Cancer (EORTC) 40891 trial [42] was conducted in Europe shortly afterward, aiming to address some of the limitations of GITSG 9173. This study similarly explored adjuvant chemoradiation using a split-course radiation regimen with concurrent 5-FU. However, these investigators included both pancreatic head and periampullary cancers, included patients with positive surgical margins, and did not provide maintenance chemotherapy after completion of radiation therapy. The study failed to demonstrate a statistically significant survival benefit overall or on subgroup analysis of patients with pancreatic head adenocarcinoma (median OS 17.1 vs. 12.6 months, *p* = 0.099) [40]. The lack of survival benefit was confirmed on long-term follow-up analysis [43]. This study was criticized for its small sample size, high dropout rate, inclusion of periampullary cancers, and lack of stratification by margin status. However, upon re-analysis using a one-sided log-rank test, Garofalo et al. found significantly improved overall survival at 2 years (37% vs. 23%, *p* = 0.049) among patients with pancreatic cancer who received adjuvant chemoradiation [44].

The conflicting results of these studies left the efficacy of adjuvant chemoradiation unclear, setting the stage for ESPAC-1. This large, multicenter study explored the role of both adjuvant chemotherapy and adjuvant chemoradiation. As discussed above, the study used a 2 × 2 factorial design: observation, chemotherapy alone (5-FU and leucovorin), chemoradiation alone (5-FU bolus with split-course radiation), or both adjuvant chemotherapy and CRT. Radiation therapy similarly consisted of a split-course regimen, while the chemotherapy regimen entailed 5-FU for 6 cycles. With respect to chemoradiation, not only did the study fail to support a survival benefit, but it suggested potential harm. Median overall survival was worse in those who received chemoradiation compared to those who did not, though this did not reach statistical significance (15.9 vs. 19.9 months, *p* = 0.05) [4]. This study was also not without criticisms, which included allowing physicians to deliver background adjuvant therapy prior to randomization, leading to one-third of the patients in the observation and chemotherapy groups also receiving radiation, lack of central review, and lack of radiation field design parameters [39]. Nevertheless, the results significantly impacted practice patterns, particularly in Europe where adjuvant chemoradiation was largely abandoned.

Following ESPAC-1, several meta-analyses attempted to clarify the question of adjuvant radiation. Stocken et al. performed an individual patient data meta-analysis of five randomized controlled trials, including the three aforementioned studies, to assess the benefit of adjuvant therapy in resected PDAC, obtaining individual patient data from all but GITSG-9173 [45]. As expected, the study found that adjuvant chemotherapy improved survival (mOS 19.0 vs. 13.5 months, *p* = 0.001). Critically, the investigators showed no benefit with adjuvant chemoradiation overall (mOS 15.8 vs. 15.2 months, *p* = 0.43). However, subgroup analysis suggested chemoradiation was more effective in patients with positive resection margins, signaling a potential complementary role for chemoradiation [45]. Khanna et al. [46] similarly performed a meta-analysis, which included the same core randomized trials (GITSG-9173, EORTC-40891, and ESPAC-1) and found improved two-year overall survival (absolute survival benefit 12%, *p* = 0.01) in those who received adjuvant chemoradiation. The diminished survival benefit of CRT over surgery alone observed in studies published after 1997, compared with earlier studies, may reflect improvements in surgical outcomes over time. When analysis was performed in the three studies published after 1997, the survival advantage was abrogated (absolute survival benefit 8%, *p* = 0.15). In 2013, Liao et al. published a network meta-analysis of adjuvant therapies for PDAC. The study included 10 published studies, including 9 randomized trials, and concluded that adjuvant chemoradiation alone provided no survival advantage over observation (HR = 0.91, CI 0.55–1.46). Even more, the addition of chemoradiation to adjuvant chemotherapy with gemcitabine or fluorouracil showed no survival advantage but increased toxic effects [47].

While individual clinical trials and meta-analyses of these trials have consistently demonstrated no significant benefit with adjuvant chemoradiation, multiple large retrospective studies have suggested the opposite. Morganti et al. conducted a multicenter retrospective pooled analysis of 955 patients assessing the benefit of adjuvant therapy in resected PDAC [48]. The study found a significant survival advantage of adjuvant chemoradiation (mOS 39.5 vs. 24.8 months, *p* < 0.001) but not adjuvant chemotherapy (mOS 29.5 vs. 43.4 months, *p* < 0.001). The retrospective nature of this study and non-mutually exclusive treatment groups inherently introduce selection bias and make these results difficult to interpret. Subsequent large database analyses exploring the impact of adjuvant radiation have suggested improved survival in those who received adjuvant radiation, particularly in high-risk patient populations with R1 resection margins or node-positive disease [49,50,51]. These studies are similarly limited by their retrospective nature and lack of comprehensive patient-level data.

In an attempt to reconcile the discrepancy in the literature regarding the clinical effect of adjuvant chemoradiation in PDAC, Ren et al. recently published the largest meta-analysis to date incorporating both RCTs and retrospective studies assessing the role of adjuvant chemoradiation in resected PDAC [52]. Nineteen studies published between 1999 and 2021 were included in their analysis. The study compared CRT to no CRT, in addition to subgroup analysis comparing CRT to observation or chemotherapy alone. The investigators found that CRT was associated with reduced odds of death at 2 years (OR 0.63, *p* < 0.001), which was consistent across subgroup analyses with observation or chemotherapy. While CRT continued to demonstrate survival benefit at 5 years when compared to observation (OR 0.67, *p* = 0.03), it was not associated with significant survival benefit when compared to chemotherapy alone (OR 0.57, *p* = 0.62). This analysis suggests improved short-term outcomes with CRT but no long-term survival benefit, consistent with the biological reality that distant metastasis, and not local recurrence, is the predominant cause of death in pancreatic cancer.

RTOG 9704 compared gemcitabine- or 5-FU-based chemoradiation for adjuvant therapy in resected pancreatic cancer. Patients were randomized to receive either gemcitabine or 5-FU for 3 weeks before and 12 weeks after CRT, which consisted of 50.4 gray (Gy) radiation in 28 fractions with concurrent daily 5-FU infusion. This study did not reveal a significant difference between gemcitabine and 5-FU in terms of overall survival. However, >70% of patients had distant recurrence, which led to the question of whether CRT should be administered after completion of adjuvant chemotherapy [7]. NRG Oncology/RTOG 0848 was designed to answer this question. The trial utilized a two-step design in which patients were first randomized to receive adjuvant chemotherapy with gemcitabine or gemcitabine plus erlotinib for five cycles. Those without evidence of disease progression following these five cycles were then randomized to receive a 6th cycle of the same chemotherapy with or without adjuvant chemoradiation, which consisted of 50.4 Gy in 28 fractions with concurrent 5-FU or capecitabine. Step 1 of the study was switched from a phase 3 to phase 2 design following publication of the LAP07 trial, which did not find benefit in the addition of erlotinib to gemcitabine for unresectable locally advanced pancreatic cancer [53]. Results from the second step of the study were recently published and showed no overall survival benefit with the addition of CRT (mOS 27.3 months with CRT vs. 31.1 months without, *p* = 0.77) but did show a trend toward improved DFS (median DFS 15.6 vs. 12.4 months, *p* = 0.089). Nodal status was found to significantly interact with treatment for both OS (*p* = 0.006) and DFS (*p* = 0.01). When accounting for nodal status, the investigators found that patients with negative nodal disease showed improvement in both OS (3 vs. 3.9 years) and DFS (1.5 vs. 2.3 years) with the addition of CRT to chemotherapy [41]. This study introduces a possible role for CRT after adjuvant chemotherapy in patients with node-negative disease. Given that the majority of the retrospective studies have shown survival benefit with CRT in patients with high-risk features and node-positive disease [48,50,51,54,55,56,57], this finding requires further investigation. It is possible that improvements in radiation regimens, quality assurance, and perioperative chemotherapy regimens may partially account for these findings. Additionally, lymph node positivity may indicate occult metastatic disease, in which case irradiating the surgical bed would likely not improve overall disease control. Another important question is the utility of radiation in the context of modern multiagent regimens like mFOLFIRINOX that may be able to achieve greater locoregional disease clearance on their own, obviating the need for additional radiation.

Currently, NCCN does not recommend CRT as a preferred adjuvant therapy but notes that it may be utilized in patients with disease features that confer a high risk for local recurrence, namely positive resection margins, while emphasizing that the role of adjuvant CRT is still being evaluated. In this setting, chemotherapy should be given prior to chemoradiation. Given the conflicting evidence, the decision to pursue adjuvant CRT should be individualized. The most recent clinical trial data (NRG/RTOG 0848) suggests that patients with favorable pathology, specifically node-negative disease, are most likely to benefit, whereas older observational data suggest the opposite, favoring high-risk subgroups such as those with positive margins or node-positive disease. Current guidelines have not yet incorporated this latest evidence. Regardless, prevailing practice maintains systemic chemotherapy as the backbone of adjuvant therapy, with CRT administered only afterward, and only if indicated. Further prospective trials are needed before any change to current guideline management can be expected.

## 5. Neoadjuvant Therapy

A discussion of adjuvant therapy for localized PDAC is not complete without acknowledging the paradigm shift towards neoadjuvant therapy. The rationale for early systemic therapy is multifactorial. Patients who receive neoadjuvant therapy are more likely to receive systemic therapy overall, as a significant proportion of patients, about 33%, will not go on to receive systemic therapy after surgery, primarily due to frailty or surgical complications [2]. Emphasizing this gap, one NCDB analysis found that receipt of neoadjuvant chemotherapy was the factor most strongly associated with timely receipt of adjuvant chemotherapy, ahead of socioeconomic factors such as median income and private insurance [58]. The neoadjuvant approach also allows for earlier treatment of micrometastatic disease, positive selection of patients appropriate for surgery, as disease progression on neoadjuvant therapy signals biologically aggressive disease, and may provide improved surgical and clinical outcomes.

One of the first randomized trials evaluating neoadjuvant therapy in PDAC was the PREOPANC-1 trial, which compared neoadjuvant gemcitabine-based chemoradiation followed by surgery and adjuvant gemcitabine to upfront surgery followed by adjuvant gemcitabine for resectable and borderline resectable PDAC [59]. The investigators found a greater likelihood of R0 resection and a lower likelihood of pathologic lymph node involvement. These superior pathologic outcomes were accompanied by improved disease-free survival (HR 0.69, *p* = 0.009) and locoregional failure-free interval (HR 0.57, *p* = 0.004) but not distant metastasis-free interval (HR 0.74, *p* = 0.07). The initial analysis showed no significant overall survival benefit with neoadjuvant therapy compared with upfront surgery (mOS 16.0 vs. 14.3 months, *p* = 0.096) [59]. Although the overall survival benefit reached statistical significance on long-term follow-up analysis (mOS 15.7 vs. 14.3 months, *p* = 0.025), subgroup analysis by resectability suggested that this was driven largely by the benefit in the borderline resectable subgroup, with no statistically significant benefit in the resectable subgroup (HR 0.79, *p* = 0.23) [60]. Notably, the resection rate in the neoadjuvant arm was numerically lower (61% vs. 72%, *p* = 0.058), reflecting disease progression during preoperative treatment, although patients in this arm received a higher cumulative chemotherapy dose [60]. Although this trial was the first to suggest a benefit of neoadjuvant therapy over upfront surgery, it was limited by its mixed resectable and borderline resectable cohort, outdated chemotherapy regimen, and unequal number of planned systemic therapy cycles between arms.

While PREOPANC-1 was ongoing, PRODIGE-24 established mFOLFIRINOX as the standard of care for adjuvant chemotherapy. Subsequently, PREOPANC-2 compared neoadjuvant FOLFIRINOX with neoadjuvant gemcitabine-based chemoradiation plus adjuvant gemcitabine for patients with resectable and borderline resectable PDAC. At a median follow-up of 42.3 months, there was no difference between the FOLFIRINOX and CRT groups in median overall survival (21.9 vs. 21.3 months, respectively; *p* = 0.32) [61]. Given that mFOLFIRINOX is superior to gemcitabine in the adjuvant setting, the equivalent outcomes between these two regimens in the neoadjuvant setting may indicate that RT can synergize with gemcitabine to deliver improved outcomes, at least in the neoadjuvant setting.

With regard to neoadjuvant chemotherapy, several meta-analyses have indicated improved overall survival when compared to adjuvant chemotherapy, although no phase 3 trials have definitively addressed this question [62,63]. The multicenter, phase 2 NORPACT-1 trial compared neoadjuvant FOLFIRINOX to upfront surgery in patients with resectable pancreatic head adenocarcinoma. While upfront surgery showed improved median overall survival on intention-to-treat analysis (38.5 vs. 25.1 months, *p* = 0.05) and numerically greater survival on per-protocol analysis (34.4 vs. 23.0 months, *p* = 0.058), the study had major methodological weaknesses [64]. Critics noted the use of full-dose FOLFIRINOX rather than modified FOLFIRINOX, which drove high toxicity and contributed to only 60% of participants completing all four cycles. Furthermore, the study’s small sample size, substantial crossover, anomalously high survival in the upfront surgery arm, and heterogeneous adjuvant therapy regimens limited its validity.

While NORPACT-1 compared neoadjuvant chemotherapy to upfront surgery in resectable PDAC, ESPAC-5 explored this question in borderline resectable disease. The study used a four-arm design, comparing upfront surgery to three different short-course neoadjuvant therapy regimens (mFOLFIRINOX, gemcitabine plus capecitabine, or capecitabine-based CRT). Although recruitment was limited to only 90 patients, the trial demonstrated improved DFS and OS in the neoadjuvant therapy arms compared with upfront surgery (1-year OS 76% for the pooled neoadjuvant therapy groups vs. 39% for upfront surgery, *p* = 0.005), with both neoadjuvant chemotherapy arms outperforming CRT [65]. Despite criticisms regarding small sample size and short follow-up duration, the study’s striking results helped to cement neoadjuvant therapy as the standard of care for borderline resectable PDAC.

The central unanswered question following these trials is whether neoadjuvant or perioperative mFOLFIRINOX is superior to upfront surgery in strictly resectable PDAC. PREOPANC-3 (NCT04927780) [66] and Alliance A021806 (NCT04340141) [67] are two ongoing clinical trials designed to answer this question. The phase 3 PACT-21 (Pancreatic Adenocarcinoma Clinical Trials-21) CASSANDRA trial (NCT04793932), whose preliminary results have recently been published, compares PAXG (cisplatin, nab-paclitaxel, capecitabine, and gemcitabine) to mFOLFIRINOX in the perioperative setting for resectable and borderline resectable PDAC [68]. The study’s 2 × 2 factorial design first randomizes patients to neoadjuvant PAXG or mFOLFIRINOX for 4 months, followed by a second randomization to an additional 2 months of the same chemotherapy, either before or after surgery. Preliminary data following the first randomization show improved event-free survival in the PAXG arm compared with the mFOLFIRINOX arm (16.0 vs. 10.2 months, *p* = 0.002). Although promising, final analysis is pending. While these ongoing trials may affect practice patterns, the current NCCN guidelines recommend neoadjuvant therapy for borderline resectable disease. In resectable disease, neoadjuvant therapy may be considered in patients with high-risk features, defined as equivocal or indeterminate imaging findings, markedly elevated CA 19-9, large primary tumors, large regional lymph nodes, excessive weight loss, and extreme pain. However, the NCCN emphasizes that neoadjuvant therapy in resectable disease is supported by limited evidence and should be conducted in a clinical trial setting [24].

## 6. Novel Therapies

Novel therapies are a significant area of research given the poor prognosis of PDAC at any stage. NCCN guidelines recommend genomic profiling of locally advanced and metastatic pancreatic cancer to identify actionable mutations [24]. PARP inhibitors represent the most established class of targeted therapy in PDAC, with olaparib demonstrating improved progression-free survival in patients with metastatic PDAC harboring germline BRCA1/2 mutations [42]. The APOLLO trial (NCT04858334) is currently underway to explore the role of adjuvant olaparib in patients with BRCA1, BRCA2, or PALB2 mutations after curative-intent surgical resection [69].

Beyond DNA damage repair, biomarker-driven therapeutic strategies in PDAC have turned to direct oncogene inhibition. Activating mutations in the KRAS oncogene are present in over 90% of patients with PDAC, most commonly G12D, G12V, and G12R [70]. Once considered “undruggable,” developments in KRAS inhibition represent a significant breakthrough in precision oncology. First-generation KRAS inhibitors are highly allele-specific, with KRAS G12C inhibitors representing the most established class. CodeBreaK 100 (NCT03600883) was the first in-human trial of a KRAS G12C inhibitor. This phase 1/2 trial assessed sotorasib in KRAS G12C-mutated advanced PDAC patients who had received at least one previous systemic therapy and produced a partial response in 8 of 38 patients, achieving a median OS of 6.9 months [71]. Similarly, KRYSTAL-1 (NCT03785249) evaluated adagrasib in a cohort of solid-tumor patients, producing a partial response in 7 of 21 patients with KRAS G12C-mutated PDAC [72]. Despite modest response rates, resistance to KRAS G12C inhibitors invariably develops. The dominant resistance patterns are secondary KRAS mutations, KRAS amplification, and reactivation of the RAS-MAPK pathway through downstream effector mutations [73]. Nongenetic mechanisms, including epithelial-to-mesenchymal transition, histologic transformation, and adaptive changes in the tumor microenvironment, further contribute to resistance [74,75]. This class is further limited by low target prevalence, as KRAS G12C is present in only 1–2% of PDAC [70]. Currently, these therapies are only FDA-approved for NSCLC and colorectal cancer. The NCCN guideline notes that this therapy may be useful in certain circumstances, specifically for KRAS G12C-mutated PDAC as a subsequent-line therapy.

More recently, second-generation pan-RAS inhibitors have emerged as a potential paradigm-altering therapy for metastatic pancreatic cancer. In a trial of previously treated metastatic PDAC, including 91.8% with KRAS G12 mutations, patients treated with daraxonrasib had significantly prolonged median OS compared to those who received chemotherapy (13.2 vs. 6.7 months, *p* < 0.001) [76]. Although data in the adjuvant setting are limited, the phase 3 RASolute 304 trial (NCT07252232) is currently comparing the RAS (ON) inhibitor, daraxonrasib, against standard of care after completion of adjuvant or neoadjuvant therapy in resected PDAC [77].

The evolution of targeted therapies has revealed their intertwined relationship with the tumor microenvironment and tumor metabolism. Oncogenic RAS signaling promotes the fibrotic, immunologically cold phenotype of PDAC, and KRAS inhibition partially reverses this, allowing immune cell infiltration. Single-agent therapy, however, is followed by acquired changes that restore an immunosuppressive microenvironment despite ongoing RAS inhibition [78].

Similarly, adaptations occur at the level of tumor metabolism. Following KRAS inhibition, PDAC cells shift toward an oxidative-phosphorylation-dependent, drug-resistant state that can be pharmacologically reversed to restore KRAS inhibitor sensitivity in preclinical models [79]. A parallel dependency on lipophagy-driven fatty acid oxidation can be co-targeted alongside KRAS, MEK, or ERK inhibition to synergistically impair tumor growth in cell lines and organoids [80]. Similar compensatory metabolic mechanisms have been described in preclinical models of lung cancer, in which DAPK2 dysfunction alters the mitochondrial cristae protein Mic60, possibly via lactylation, increasing mitochondrial metabolism in cells with EGFR-TKI resistance and enhanced metastatic potential [81]. In PDAC, globally increased protein lactylation is likewise associated with an immunosuppressive microenvironment and poor response to checkpoint blockade [82]. These metabolic alterations, however, also create new vulnerabilities. Hafner et al. showed that dual inhibition of SHP2 and MEK1/2 stimulates compensatory mitochondrial remodeling and disrupts reactive oxygen species homeostasis, leading to reliance on the lipid hydroperoxidase GPX4. Concurrent inhibition of GPX4 induced ferroptosis and suppressed tumor progression in vivo [83].

In a similar vein, inhibition of hyperactive focal adhesion kinase (FAK) reduces fibrosis and increases immune cell infiltration in preclinical models, but prolonged FAK inhibition results in compensatory growth signaling, requiring the addition of chemotherapy or targeted agents to achieve tumor regression [84,85]. Immunotherapy, historically ineffective in PDAC outside the small subset of mismatch repair-deficient tumors, is showing renewed promise when incorporated into multiagent regimens [86,87]. At the cellular level, the efficacy of post-operative, immune-based therapies is constrained by tumor recognition. In PDAC, MHC-1 is selectively captured by the autophagy receptor NBR1 and routed for lysosomal degradation, preventing recognition by immune cells. Inhibition of autophagy or lysosomal function restores surface MHC-1, enhances antigen presentation, and synergizes with checkpoint blockade in preclinical models [88]. Antigen display alone is not sufficient, however. Immune recognition requires antigens of sufficient quality. Long-term PDAC survivors are distinguished by neoantigen quality and T cell infiltration rather than by neoantigen burden. Recurrent tumors in these patients have been shown to lack these high-quality neoantigens [89]. Coupled with the desmoplastic microenvironment that spatially excludes effector cells, immune evasion in PDAC is multifaceted, achieved through multiple complementary mechanisms. Consequently, any therapy directed at a single mechanism will be undermined by the others, necessitating a correspondingly multifaceted intervention.

Other novel therapies have explored device-based strategies, though these remain in early investigational stages. One approach uses depots implanted into the surgical bed at the time of surgery to release immunostimulatory payloads over time, based on the rationale that the surgical bed is a site of residual disease that is concurrently subject to surgery-induced immunosuppression [90]. Other investigators have explored the use of hollow mesoporous carbon nanoparticles, which generate reactive oxygen species when exposed to ultrasound to directly induce cell death. Loading these particles with immune checkpoint inhibitors enhanced the therapeutic effect [91].

Taken together, these findings argue that durable treatment response will require rationally designed, multiagent regimens that pair targeted therapies with treatments directed at the stromal, immune, or metabolic adaptations it provokes. As therapies approach clinical validation, the development of predetermined response thresholds to guide therapeutic adaptation may require consideration. Current management incorporates serial assessments of imaging, basic laboratory evaluation, clinical status, and treatment-related toxicity; yet, these measures are not integrated into prospectively defined quantitative thresholds that determine when to continue, intensify, de-escalate, or change therapy. Emerging biomarkers, including ctDNA, may provide additional indicators that more reliably reflect treatment response to facilitate such an approach. Wu et al. demonstrated, using a non-smooth mathematical model of tumor-immune dynamics, that the definition and timing of treatment thresholds can substantially influence therapeutic outcomes, and strategies incorporating multiple thresholds may optimize disease control while limiting adverse effects [92]. Although this framework has not been validated in pancreatic cancer, it provides a conceptual basis for developing response-adaptive treatment algorithms that may enable treatment to be individualized according to longitudinal measures of tumor and host response.

## 7. Recurrence

Despite multimodal therapy, survival following curative-intent resection remains poor, with 5-year overall survival ranging from 17% in real-world cohorts to 30–47% in modern trial populations [2,14]. Most recurrences occur within two years of surgery and consist largely of distant metastases, particularly to the liver, although isolated local recurrence is seen in up to 24% of patients [60,93]. This preponderance of early distant recurrence indicates that clinically occult micrometastatic disease is likely present at the time of surgery in many patients. It is partially on this basis that focus has shifted towards administering neoadjuvant therapy, rather than adjuvant therapy, to address occult micrometastatic disease earlier [94,95].

Although NCCN guidelines note that neoadjuvant therapy can be considered in resectable disease with high-risk features, explicit criteria remain undefined. As discussed above, PREOPANC-1 showed improved pathologic outcomes with greater R0 resection rates and decreased pathologic lymph node involvement, perineural invasion, and venous invasion in the neoadjuvant CRT arm. While long-term analysis showed an overall survival benefit with neoadjuvant therapy, the absolute benefit was modest (15.7 vs. 14.3 months), and subgroup analysis showed no significant benefit in those with resectable disease.

NORPACT-1 similarly compared neoadjuvant FOLFIRINOX to upfront surgery followed by adjuvant chemotherapy. In contrast to PREOPANC-1, the investigators found worse OS in the neoadjuvant arm at 18 months (60% vs. 73%, *p* = 0.032) and shorter median progression-free survival (11.9 vs. 16.2 months, *p* = 0.22) despite a greater R0 resection rate (56% vs. 39%, *p* = 0.018) and similar overall resection rates [64]. PREOPANC-2 subsequently compared neoadjuvant FOLFIRINOX to neoadjuvant gemcitabine-based CRT and found no significant difference between arms in median OS (21.9 vs. 21.3 months for FOLFIRINOX and CRT, respectively, *p* = 0.32) or 1- and 3-year overall survival. Similarly, serious adverse events and major surgical complications were comparable [61]. Pathologic node-negative resection was more frequent following CRT, but R0 resection and overall resection rates were comparable [61].

Taken together, these studies suggest that margin and nodal status are poor surrogates for survival in the neoadjuvant setting, as acknowledged by the authors of PREOPANC-2. A recent meta-analysis of nine trials in resected disease confirms this dissociation: neoadjuvant therapy significantly improved event-free survival (HR 0.77, 95% CI 0.65–0.90), reduced non-curative exploration, and nearly doubled the likelihood of pN0 histology, but produced only a non-significant trend toward improved overall survival (HR 0.85, 95% CI 0.68–1.05) [96].

This pattern of improved locoregional control and pathologic endpoints without a commensurate survival benefit is what would be predicted by a recurrence pattern dominated by distant relapse, and it is consistent with the observation that neoadjuvant chemotherapy does not alter the distribution of initial recurrence, which remains predominantly distant regardless of treatment or margin status [97]. Nonetheless, clinical benefit in the neoadjuvant approach remains; receipt of neoadjuvant chemotherapy provides earlier treatment of occult micrometastatic disease, a higher probability of receiving systemic therapy overall, as a significant proportion of patients never receive chemotherapy following surgery, and selection against surgical futility in patients with biologically aggressive disease [2,61]. Emerging biomarkers are under investigation to identify patients most likely to benefit from neoadjuvant chemotherapy, including circulating tumor DNA, although these have not been validated for routine clinical use [98,99].

Accordingly, the decision to pursue neoadjuvant therapy in resectable disease requires individualized assessment at this time. Although NCCN lists neoadjuvant therapy as an option in resectable PDAC, the decision ultimately remains at clinician discretion. The ongoing ALLIANCE A021806 (NCT04340141) and PREOPANC-3 (NCT04927780) trials are expected to provide answers to this question.

## 8. Biomarkers

The novel biomarkers used in the management of PDAC patients are worth mentioning. A major distinction must be drawn between predictive biomarkers, which identify differential response to a specific agent and therefore necessarily require a statistically significant treatment-biomarker interaction, and prognostic biomarkers, which stratify recurrence or survival risk independent of the treatment administered. The serum marker carbohydrate antigen 19-9 (CA 19-9), for example, is widely used for both prognosis and disease monitoring. As discussed above, ctDNA is a prognostic biomarker. Both pre- and postoperative ctDNA positivity are strongly associated with inferior disease-free and overall survival, although no prospective validation has demonstrated its utility in clinical decision-making [100].

Similarly, transcriptomic signatures, based primarily on GATA6 expression, have consistently demonstrated prognostic value. The widely used Moffitt classification characterizes PDAC as basal-like or classical, corresponding to GATA6-low and GATA6-high, respectively [101]. The classical subtype is strongly associated with improved survival, although it has not been validated as a predictive biomarker [102]. In contrast, one retrospective analysis demonstrated improved OS for classical subtype tumors treated with FOLFIRINOX versus gemcitabine plus nab-paclitaxel (GnP) (mOS 12.9 vs. 7.6 months for FOLFIRINOX and GnP, respectively; *p* < 0.001) [103]. The PASS-01 trial (NCT04469556), a phase 2 randomized trial, showed the opposite (mOS 9.7 vs. 13.9 months for FOLFIRINOX and GnP, respectively; *p* = 0.047) [104]. Given this discrepancy, this classification is unable to be used as a predictive biomarker. The lack of predictive validation, in conjunction with the ability of tumors to shift subtypes, limits its clinical utility, and its use is largely confined to research settings [105].

Homologous recombination deficiency (HRD), particularly biallelic BRCA1/2 or PALB2 mutations, is one of the few validated predictive biomarkers. HRD status conferred a significant PFS benefit in patients receiving first-line platinum-based therapy (HR 0.44, *p* < 0.01) but not in those receiving non-platinum therapy (HR 1.42, *p* = 0.23) [106]. Clinically, HRD is used to predict platinum-based chemotherapy sensitivity and guide treatment selection, determine eligibility for PARP inhibitors, and, because most relevant mutations are germline, trigger subsequent testing in family members. Cisplatin plus gemcitabine is listed as a preferred regimen only for known BRCA1/2 or PALB2 mutations in the NCCN guidelines for the neoadjuvant, locally advanced, and metastatic settings. Following the POLO trial (NCT02184195), which demonstrated that BRCA-mutated metastatic PDAC patients who had not progressed after 16 weeks of platinum-based therapy had improved median PFS on maintenance olaparib, a PARP inhibitor, the FDA approved olaparib as maintenance therapy after platinum-based chemotherapy for patients with germline BRCA-mutated metastatic PDAC [42].

Although HRD is a commonly utilized predictive biomarker in PDAC, hENT1 expression, a plasma membrane transporter responsible for gemcitabine uptake, is the historic prototype of a clinically validated predictive biomarker. High expression is associated with prolonged survival in gemcitabine-treated but not fluorouracil-treated patients in retrospective analyses of patient samples from RTOG9701 [107] and later ESPAC-3 [108]. Although high hENT1 has consistently predicted favorable response to gemcitabine and gemcitabine-based regimens (albeit in retrospective correlative analyses) [109,110,111], it has failed to reach widespread use because its predictive value is antibody-dependent, requiring an antibody that is not commercially available [112].

In contrast to the prognostic and predictive biomarkers discussed above, DPYD and UGT1A1 are used to predict toxicity. DPYD encodes dihydropyrimidine dehydrogenase (DPD), which inactivates 5-fluorouracil. Loss-of-function variants result in drug accumulation and significant toxicity. Four pathogenic variants are well-characterized, and carriers have up to a 25-fold elevated risk of treatment-related death. Poor metabolizers should receive an alternative agent, whereas intermediate metabolizers should receive a 25–50% dose reduction with subsequent titration guided by tolerance [113]. Similarly, UGT1A1 glucuronidates SN-38, the active metabolite of irinotecan, inactivating the drug. Carriers of loss-of-function variants are at elevated risk for treatment-related toxicity [114]. These genes have both been prospectively validated in single-arm implementation studies [115,116].

Finally, as discussed previously, post-hoc subgroup analysis from the PRODIGE 24 trial demonstrated a possible association between KRAS-wild-type tumors and poor response to mFOLFIRINOX [34]. While potentially useful clinically, this association has not been validated and requires further exploration, including prospective validation, prior to clinical implementation. This biomarker should, therefore, be regarded solely as hypothesis-generating at this time.

Collectively, biomarker development in PDAC remains at an early stage relative to other solid tumors. Presently, only HRD has achieved validation as a clinically actionable predictive biomarker, guiding treatment selection, while DPYD and UGT1A1 serve adjacent purposes of predicting treatment-related toxicity. The remaining biomarkers discussed have yet to demonstrate validated clinical utility. The promise of biomarker-directed treatment, therefore, requires further prospective validation and the identification of novel predictive, rather than prognostic, biomarkers.

## 9. Standard of Care

Current standard of care for adjuvant therapy is guided by NCCN guidelines. For resected PDAC, those who have not received prior systemic therapy are recommended to receive adjuvant chemotherapy +/− chemoradiation. Preferred regimens are mFOLFIRINOX or gemcitabine plus capecitabine, with mFOLFIRINOX reserved for patients with good performance status (ECOG 0–1). Radiation has a role in certain clinical scenarios. NCCN notes that radiation may be considered after surgery in patients with high-risk features, such as positive resection margins. In patients who have received neoadjuvant therapy, adjuvant treatment should be guided by response to neoadjuvant treatment and clinical characteristics [24].

## 10. Conclusions

Pancreatic ductal adenocarcinoma remains a highly lethal disease even after curative-intent resection. The role of adjuvant therapy in the treatment of resected pancreatic cancer has been the standard of care for the past two decades, owing to a multitude of clinical trials demonstrating its survival benefits. While the preferred regimen has evolved over time, chemotherapy in the adjuvant setting has continued to demonstrate effectiveness, whereas the role of chemoradiation has been debated. More recently, the treatment paradigm has shifted towards perioperative or total neoadjuvant systemic therapy over upfront surgery, though this has yet to be reflected in clinical treatment guidelines due to ongoing clinical trials seeking to definitively assess the clinical benefit.

While significant advances have been made in our understanding of the pathophysiology of pancreatic cancer and optimal treatments, outcomes remain poor and recurrence common. Ongoing research into targeted therapies based on genomic profiling, novel immunotherapies, and rationally developed combination regimens will continue to refine the role of adjuvant therapy. However, much of this body of exploration remains preclinical and requires further validation and risk assessment prior to utilization in clinical populations. Nonetheless, these early findings are promising and may ultimately improve our management of this disease.

## Figures and Tables

**Table 1:** Landmark clinical trials exploring adjuvant chemotherapy for resected pancreatic ductal adenocarcinoma.

Trial	Author, Year	N	Regimen	5-Year Survival	Median OS (Months)
ESPAC-1 [4]	Neoptolemos, 2004	5-FU—238 Observation—235	5-FU—425 mg/m^2^ + folinic acid 20 mg/m^2^ daily for 5 days; monthly for 6 months	5-FU—21% Obs—8%	5-FU—20.1 Obs—15.5 (*p* = 0.0005)
CONKO-1 [5]	Oettle, 2013	Gemcitabine—179 Observation—175	Gemcitabine—1000 mg/m^2^ on days 1, 8, and 15 every 4 weeks for 6 cycles	Gemcitabine—20.7% Obs—10.4%	Gemcitabine—22.8 Obs—20.2 (*p* = 0.01)
JSAP-02 [6]	Ueno, 2009	Gemcitabine—58 Observation—60	Gemcitabine—1000 mg/m^2^ on days 1, 8, and 15 every 4 weeks for 3 cycles	N/A	Gemcitabine—22.3 Obs—18.4 (*p* = 0.19)
RTOG-9704 [7]	Regine, 2011	CRT + 5-FU—230 CRT + Gemcitabine—221	5-FU—250 mg/m^2^ daily for 3 weeks, then CRT, followed by 5-FU 250 mg/m^2^ daily for 4 weeks with 2-week break, repeated for 2 cycles Gemcitabine—1000 mg/m^2^ once weekly for 3 weeks, then CRT, followed by gemcitabine 1000 mg/m^2^ once weekly for 3 weeks with 1-week break for 3 cycles CRT—50.4 Gy in 28 fractions, 5 days a week with concurrent 5-FU 250 mg/m^2^ daily	CRT + 5-FU—18% CRT + Gemcitabine—22%	CRT + 5-FU—17.1 CRT + Gemcitabine—20.5 (*p* = 0.08)
ESPAC-3 [8]	Neoptolemos, 2010	5-FU—551 Gemcitabine—537	5-FU—425 mg/m^2^ plus folinic acid 20 mg/m^2^ on days 1–5 every 28 days for 6 cycles Gemcitabine—1000 mg/m^2^ on days 1, 8, and 15 every 4 weeks for 6 cycles	5-FU—15.9% Gemcitabine—17.5% [9]	5-FU—23.0 Gemcitabine—23.6 (*p* = 0.39)
JASPAC-01 [10]	Uesaka, 2016	Gemcitabine—190 S-1—187	Gemcitabine—1000 mg/m^2^ IV on days 1, 8, and 15 every 4 weeks for 6 cycles S-1—40–60 mg twice daily for 28 days followed by 14-day rest, every 6 weeks for 4 cycles	Gemcitabine—24.4% S-1—44.1%	Gemcitabine—25.5 S-1—46.5 (*p* < 0.0001)
CONKO-005 [11]	Sinn, 2017	Gemcitabine—217 Gemcitabine + erlotinib—219	Gemcitabine—1000 mg/m^2^ IV on days 1, 8, and 15 every 4 weeks for 6 cycles Gemcitabine + erlotinib—same gemcitabine regimen plus erlotinib 100 mg orally daily, for 6 cycles	Gemcitabine—20% Gemcitabine + erlotinib—25%	Gemcitabine—26.5 Gemcitabine + erlotinib—24.5 (*p* = 0.61)
APACT [12]	Tempero, 2023	Gemcitabine—434 Nab-paclitaxel + gemcitabine—432	Gemcitabine—1000 mg/m^2^ IV on days 1, 8, and 15 every 4 weeks for 6 cycles Nab-paclitaxel + gemcitabine—same gemcitabine regimen plus nab-paclitaxel 125 mg/m^2^ on days 1, 8, and 15 every 4 weeks for 6 cycles	Gemcitabine—31% Nab-paclitaxel + gemcitabine—38%	Gemcitabine—37.7 Nab-paclitaxel + gemcitabine—41.8 (*p* = 0.009)
ESPAC-4 [13]	Palmer, 2025	Gemcitabine—366 Gemcitabine + Capecitabine—364	Gemcitabine—1000 mg/m^2^ IV on days 1, 8, and 15 every 4 weeks for 6 cycles Gemcitabine + Capecitabine—same gemcitabine regimen plus capecitabine 1660 mg/m^2^/day oral for 21 days followed by 7-day rest, for 6 cycles	Gemcitabine—25% Gemcitabine + Capecitabine—32%	Gemcitabine—28.4 Gemcitabine + Capecitabine—31.6 (*p* = 0.03)
PRODIGE-24/CCTG PA-6 [14]	Conroy, 2022	Gemcitabine—246 mFOLFIRINOX—247	Gemcitabine—1000 mg/m^2^ IV on days 1, 8, and 15 every 4 weeks for 6 cycles mFOLFIRINOX—Oxaliplatin 85 mg/m^2^, leucovorin 400 mg/m^2^, irinotecan 150 mg/m^2^, 5-FU 2400 mg/m^2^, over 46 h every 14 days for 12 cycles	Gemcitabine—31.4% mFOLFIRINOX—43.2%	Gemcitabine—35.5 mFOLFIRINOX—53.5 (*p* = 0.001)

**Table 2:** Landmark clinical trials exploring adjuvant chemoradiation therapy for resected pancreatic ductal adenocarcinoma.

Trial	Author, Year	N	Regimen	5-Year Survival	Median OS (Months)
GITSG-9173 [38]	Kalser, 1985	CRT—21 Observation—22	40 Gy in two courses of 20 Gy each with a 2-week break; concurrent 5-FU 500 mg/m^2^ IV for three consecutive days with each course of radiation followed by weekly 5-FU for up to 2 years.	CRT—18% Obs—8%	CRT—21 Obs—10.9 (*p* = 0.03)
EORTC-40891 [40]	Klinkenbijl, 1999	CRT—104 (60 with pancreatic head cancer) Observation—103 (54 with pancreatic head cancer)	40 Gy in two courses of 20 Gy each with a 2-week break; concurrent 5-FU 500 mg/m^2^ IV for three consecutive days with each course of radiation	NA	Pancreatic Head Cancer Subgroup CRT—17.1 Obs—12.6 (*p* = 0.099)
ESPAC-1 [4]	Neoptolemos, 2004	CRT—175 No CRT—178	40 Gy in two courses of 20 Gy each with a 2-week break; concurrent 5-FU 500 mg/m^2^ IV for three consecutive days with each course of radiation	CRT—10% No CRT—20%	CRT—15.9 No CRT—17.9 (*p* = 0.05)
RTOG-9704 [7]	Regine, 2011	CRT + 5-FU—221 CRT + Gemcitabine—230	50.4 Gy in 28 fractions, 5 days a week with concurrent continuous 5-FU infusion	CRT + 5-FU—18% CRT + Gemcitabine—22%	CRT + 5-FU—17.1 CRT + Gemcitabine—20.5
RTOG-0848 [41]	Abrams, 2026	Chemotherapy—174 Chemotherapy + CRT—180	50.4 Gy in 28 fractions, 5 days per week, with concurrent fluoropyrimidine-based chemotherapy (Capecitabine or 5-FU)	CRT—26%, 41% No CRT—18% Node-Negative Disease CRT—41% No CRT—26%	CRT—27.6 No CRT—31.2 (*p* = 0.38)

## Data Availability

No new data were created or analyzed in this study. Data sharing is not applicable to this article.
